# What are the key leadership competencies required by medical school deans in Uganda? A qualitative cross-sectional study

**DOI:** 10.4314/ahs.v21i4.54

**Published:** 2021-12

**Authors:** Patrick Kyamanywa, Peter Redding

**Affiliations:** 1 Deputy Vice Chancellor of the Western Campus at Kampala International University and Professor of surgery in the Faculty of Clinical Medicine and Dentistry, Kampala International University Western Campus, Uganda; 2 Freelance academic consultant at Robert Kennedy College, Zurich, Switzerland. peter.redding@rkc.edu

**Keywords:** Competencies, effective leadership, medical schools, Dean

## Abstract

**Background:**

Effective leadership is vital for organizational growth and sustainability. Globally, medical schools are faced with leadership challenges due to the pace of globalization, technological advances, reduced funding and changed funding cycles, increasing student enrolment, demands of accreditation, academic collaboration, innovations and research. This makes identification and selection for the right leadership competencies a priority.

**Objectives:**

To investigate the key leadership competencies required by deans of medical schools in Uganda.

**Method:**

A qualitative study using semi-structured interviews with the current deans and purposively selected former deans of medical schools in Uganda was conducted between March and June, 2020. We analysed the data using Grounded theory.

**Results:**

Thirteen (13) deans (9 of the 12 current deans and 4 former deans) participated in the study. We established ten (10) key roles of a dean of a medical school categorised as academic leadership, administrative leadership and professional leadership. Eleven (11) key competencies were identified as necessary for effective leadership of medical schools in Uganda, and categorized as personality-related competencies, organizational management competencies and medical/health expertise.

**Conclusions:**

A dean of a medical school in Uganda should possess a combination of personality, medical expertise, health professions training and organizational management competencies and have training in leadership, financial and resources management.

## Introduction

Leadership is “the ability of an individual to influence, motivate and enable others to contribute toward the effectiveness and success of the organization of which they are members” 1. Although it is argued that, effective leadership of educational institutions differs from that in other organizations^2^,^3^, institutions of higher learning, stand and fall on their leadership^4^,^5^; making the need to have the right set of leadership competencies to inform leadership identification, selection, development and assessment important. In examining the role of leadership in university performance in Uganda, Muriisa^6^ argues that “the role of leadership has been overlooked yet they occupy a central role in the performance of the university.”

Medical schools tend to appoint deans from amongst the top academic hierarchy on a seniority and collegial basis often with the ‘first among equals’ being appointed to the role^7^. The assumption is that seniority and academic excellence correlate with effective leadership competencies^2^,^8^,^9^. However, Altbach^10^ observes that, “ the traditional...pattern of control by senior professors... elected from among their ranks..., is perhaps no longer practical, in light of the myriad skills demanded of an effective university leader.”

Leadership competencies are, “sets of behaviours that are instrumental in the delivery of desired results or outcomes”^13^ and encompass knowledge, skills, attitudes, behaviours^8^,^14^,^15^, emotional intelligence and experience^15^,^16^,^17^ necessary for effective performance as a leader.

Globally, medical schools like other organizations are faced with leadership challenges^19^,^20^. The field of medical education in the 21^st^ Century is undergoing tremendous changes occasioned by globalization, technological advances, reduced funding and changed funding cycles, increasing student enrolment, demands of accreditation, academic collaboration, innovations and research^7^. The current Coronavirus (COVID-19) pandemic has also forced a disruption in higher education changing the way we engage the students, faculty and staff^21^,^22^, hence, the need to identify and select for the right competencies for effective leadership^8^,^23^,^24^.

Uganda currently has twelve medical schools, six public and six private^25^. The recruitment and appointment of deans is done differently in each one of these. In public medical schools, deans are internally elected by the faculty members from among the senior academic staff, and appointed by the University Council^26^. The elected dean holds office for a period of 4 years and can only serve for a maximum of 8 years. However a dean may also be directly appointed by the Vice Chancellor in case of a new medical school in a public university^26^. In private schools deans are sourced and appointed by the proprietors or the university leadership. There are no national or regional guidelines specifying the roles and competencies of medical school deans.

This study was informed by leadership theory and leadership competence models^5^,^27^. The concepts of leadership competencies and competency models arise from the behavioural and situational leadership theories^28^. Leadership competencies are a response to the rapidly changing job environments and the need to develop leaders capable of working in dynamic situations^29^.

We aimed at investigating the key leadership competencies required by deans of medical schools in Uganda. This is the first study investigating the key leadership competencies required by deans of medical schools in Uganda. It is hoped that the findings of this study will inform the selection, appointment, appraisal and development of deans to effectively lead the medical schools in Uganda and the region through the highly dynamic 21^st^ Century environment.

## Method

This was a cross-sectional qualitative survey design drawing on Grounded theory^30^. We applied interpretive and subjective philosophy, acknowledging the deans' social experiences, meanings and interpretations in the conduct of their roles and their ability to develop an in-depth understanding about the competencies for leadership of medical scool in Uganda. We also used an inductive approach relying on the ability of the deans to reflect on their lived experiences, interpretations and perceptions, so as to identify a set of key competencies necessary for deans to offer effective leadership of medical schools in Uganda. Due to the Coronavirus pandemic and restrictions on human contact, telephone interviews were used in preference to internet-based platforms because of the more reliable connection offered by telephones when working from home during the lockdown in the Ugandan setting.

### Participants

We invited the participation of all twelve current deans and a purposively selected number of former deans drawn from private and public medical schools in Uganda. We purposively sampled the former deans on grounds of their experience (more than 3 years out of office as deans; appointment to higher offices such as college principal, after the deanship; and championing major changes in the medical schools during their tenure), and the different characteristics of their medical schools – whether public or private, to achieve deeper insights and reach theoretical saturation^30^.

### Data collection

We developed an interview guide with eleven (11) questions requiring the participants to describe their roles as deans, reflect on a typical week in the office of the dean, the key events reminiscent of successes, challenges faced, and leadership approaches applied in the conduct of their jobs. Finally, participants identified and discussed what they each perceived as key competencies used or necessary for effective performance as a dean of their medical school, and their leadership training needs.

We used a flexible data collection strategy with semi-structured open-ended interviews lasting between 45 minutes to one hour. All interviews were recorded using a digital voice recorder. The order of the interviews and assigning numbers to the current and former deans was done after confirming the interview date and time with the respective dean. For example the first interview with a current dean was labelled ‘CD1’, and with a former dean, ‘FD1’. Current and former deans were interviewed concurrently.

### Data analysis

Drawing on Grounded theory approaches to data analysis^30^,^31^ we immediately transcribed all audio-recorded interview data and notes after every interview session. Initial open coding was done as a back and forth process, using an inductive approach to identify codes for the roles of a dean and then codes for the leadership competencies from the thirteen transcribed interviews. We identified a total of 27 open codes in the open coding stage for the roles of a dean and 51 open codes for the leadership competencies.

Subsequently, focused coding was done grouping the similar open codes and eliminating duplications to generate categories informed by ideas from the interview and existing literature^30^,^31^. A total of 10 focused codes were identified for the roles of a dean and 11 focused codes for the leadership competencies. We then applied the identified codes to the transcripts, marking out on the transcript the relevant lines fitting the codes.

We further analysed the focused codes for relationships to identify principal categories^30^. Three principal categories of roles: academic roles, administrative roles and professional roles, were identified, and three for the leadership competencies: personality, training and organizational skills. We used the results of the analysis to inform the development of a Grounded theory on the leadership competencies for effective leadership of medical schools in Uganda.

## Results

We approached all the 12 current deans and 6 former deans (3 each from public and private medical schools) to solicit their participation in the study. Thirteen (13) deans participated in the study. These included nine (75%) of the current deans (CD) and four (67%) of the purposively selected former deans (FD). Two of the current deans (15%) were female. Three (25%) current and 2 former deans did not respond when contacted. The participants' duration in office ranged from 4 months to 12 years with an average of 4 years and a combined experience of 54 years as medical school deans. We realised data saturation after the fifth interview, having interviewed 3 current deans and 2 former deans.

We present the results of the analysis starting with the identified roles of a medical school dean, and followed by the identified key leadership competencies as generated from the focused codes consolidated into principal categories. Exemplary quotes from the transcribed interviews are used to illustrate the generated codes. For the confidentiality of the participants, current deans are abbreviated as “CD” and assigned serial numbers in the order of the interviews (1–9), while former deans are labelled as “FD” and assigned serial numbers in the order of the interviews (1–4).

### Role of a Dean of a Medical School

All the participants acknowledged that the office of the dean simultaneously handles academic, management and administrative roles. Some of the participants maintained a teaching and/or clinical schedule in addition to their dean responsibilities. One of the participants described it as:

”...one where you can partly adequately plan for your week...although frequently you get interrupted by things you never planned for because you are not the ultimate authority of yourself. Some of your time is controlled by other people and the environment...”FD4

Ten (10) major roles of a dean of a medical school were identified. The major roles were consolidated into three (3) principal categories; academic leadership, administrative leadership and professional leadership as shown in table 1.

**Table 1:** Principal categories from the focused codes for roles of deans

Principal categories	Focused codes
**Academic leadership**	1. Academic leadership 2. Research leadership
**Administrative leadership**	3. Strategic leadership 4. Capacity development 5. Financial and resources management 6. Communication and collaboration 7. Quality assurance
**Professional leadership**	8. Advisor 9. Role model 10. Professional leadership

One participant said:

“*The role of a dean ...is to give the school a strategic leader ship and to provide management, and to be able to instil some future direction and then to manage the resources in cluding funds...*”FD2

In handling communication and collaboration, the dean also plays an advocacy role as observed by another participant:

“*...at the same time the role of a medical school dean is to be an advocate for the institution and the profession, for the education of health professionals in an environment with many competing needs...*”FD4

A dean provides professional leadership through being a role model and offering clinical, health, scientific and educational expertise as an advisor and practitioner. Participant (CD9) observed that a dean “...is an advisor to the university...” and another said a dean is “... called upon to provide expertise, to contribute to discussions and to plan for the health environment of the country...”FD4

Furthermore, the dean is expected to be a role model for the students, faculty, and staff and in the community of practice as observed by one participant,

“*...it is my role...to ensure that I lead by example to up hold the vision and the mission and the core values of the faculty because ...I occupy the highest office in the faculty ...*”CD4

### Leadership Competencies for Effective Deanship in Uganda

Eleven (11) key competencies were identified for effective deanship of a medical school in Uganda. The eleven competencies were consolidated into three principal categories; personality-related competencies, education/training related competencies and organizational management skills as shown in Table 2.

**Table 2:** Principal categories from the Focused codes for leadership competencies

Principal categories	Focused codes
**Personality**	1. Personality 2. Open to Learning and self- improvement
**Education/training**	3. Medical expert 4. Medical educator 5. Research skills
**Organizational skills**	6. People management 7. Work ethics 8. Financial and resources management 9. Communication and collaboration 10. Strategic management 11. Change management

### Personality-related Competencies

We identified two (2) personality-related competencies; 1- Personality behavioural attributes (honesty, integrity, humility, trustworthiness, confidence, diplomacy, patience, maturity and empathy); and 2- Openness to learning and self-improvement. According to one current dean:

“*...should be a type of person who is ready to accept that you are not always correct and therefore should listen to others' opinions...be able to accept to do things in an acceptable way for all stakeholders... willing to strictly adhere to policies, standards and protocols.*” CD5

Furthermore, a dean should;

“*... [be] frank. I want to communicate with honesty and be frank with people...a dean cannot be effective if they do not appreciate the position they are in as people with authority and in leadership...*” CD6

Another participant observed that:

“*...having the pulse of everybody is difficult but it is essen tial...knowing how everybody is fairing...it is very important to know that everybody is treated equally and whoever falls off is brought back without being abused...and people who do well should be applauded ...in public...*” FD3

Regarding openness to learning and self-improvement, CD9 remarked that; “*...one has to keep oneself up to date...* ”and FD4 advised to “*...learn new skills...and open up your thinking and begin to appreciate that you need social sciences and interactions of professionals...*”

### Education/Training related Competencies

Three (3) education/training competencies were identified. All the participants stated that it was important for a dean to be: 1- a medical or health expert with postgraduate training; 2 - competent in medical or health professional education and pedagogy; and 3- have research skills.

As observed by CD1;

“*A dean should be a medical educator. Medical educa tion is a bit more complex than other forms of formal education and so ... techniques in medical education would be very useful for the dean to have in order to run and manage the programs...*”

Another participant said;

“*They should be knowledgeable about biomedical and clin ical training and ... should have the relevant training... have a masters training to take the students and the faculty they lead to the level where the university wants them to be...should be able to participate in research, evidenced by publications, grants and collaborations...*” CD3

The above perceptions were further enforced by FD4 who asserted that;

“*...the medical school dean has to be part of the health professional educator scheme because you are training health professionals and therefore need to pay attention to health professional development and education*.”

### Organizational Competencies

Six (6) competencies were categorized as organizational competencies: 1-People management skills, 2- work ethics, 3- financial and resources management skills, 4-communication and collaboration, 5- strategic management and 6- change management.

### People management skills

Participants stressed the importance of team building, transparency, mentoring, delegating and empowering others as key skills required by a dean and especially in building capacity. Conflict management was another special skill recognized by most of the participants. Participant CD6 stated that,

“*...a dean...should appreciate teamwork, and ... ensure there is teamwork and ...give room to other appointed heads of departments, chairs of committees and other staff to do their work...*”

Furthermore, a dean should;

“*...get a team and build their capacity for health professions education...therefore setting the stage for advancement...*”

### Work ethics

This category included multi-tasking skills, resilience, commitment, personal/self-management, having passion for the job, flexibility, consistency and quality assurance, attention to detail, being hard working and results oriented,. Participant CD5 noted that;

“*...you have to be somebody who is able to multi-task, being able to wear very many different types of hats in a meaningful way in order to be able to succeed because of the various roles you are expected to handle at any particular moment*.”

Another, CD2 observed that, “*... time management and distribution because there are many activities...is a very important skill...*”and CD7 said, “*...because you are expected to supervise, guide, support and evaluate what people are doing... you must have skills in quality assurance...*” Participant CD8 observed that, “*...someone has to be...hardworking, able to absorb pressure, work under stress...*”

All participants stressed the importance of Financial and resources management skills. One participant CD 7 stressed that, “*...you need to know about how to run an institution especially financial management...and if you are unable to handle you can be in big trouble...*” Participant FD4 also remarked that, “*...the medical school dean must be involved in resource mobilization...not just money...needs to be very strategic in getting the human resource that is required at different levels...*”

### Communication and Collaboration skills

These were also considered very important by all participants. Under this subcategory were skills such as communication, collaboration, advocacy, networking, persuasion, negotiation, and listening skills. Participant CD1 stressed the skill of “*...building dialogue, wide consultation and involvement of several stakeholders...*”

### Strategic management

The ability to create and communicate a compelling vision was identified by all the participants. A dean has to be visionary, creative, strategic and optimistic. A dean, “*...should be fairly ambitious...able to see things five to ten years ahead...and try to benchmark with bigger schools...*”FD2. According to CD3, a dean should be, “*...having a vision for the school and keeping track of the vision...*”

### Change management

Every participant reported having been involved in managing a change process and so found change management skills to be essential. According to FD4, “*... being a change agent is one of the most important...the ability and skills to effectively manage in an institution...*”

**Figure 1:**
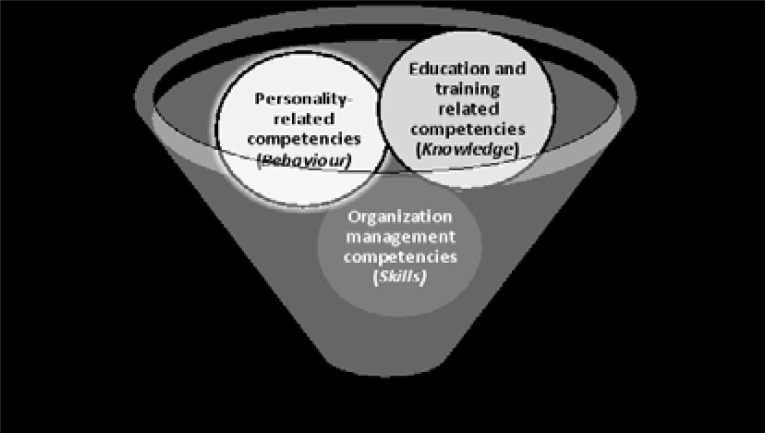
Summary of key leadership competency categories for effective leadership of a medical school

Figure 1 shows a framework developed from the study findings to summarize the key competencies for effective leadership of a medical school in Uganda.

All the participants observed the need for a deliberate and systematic effort to train current and prospective deans in leadership, financial and resources management, so as to enhance the deans' performance and in turn the growth and competitive advantage of their medical schools.

## Discussion

In general universities in Uganda face leadership challenges^6^,^32^,^33^. No studies have been done to examine the leadership of medical schools in Uganda. The aim of our study was to investigate the key competencies for effective leadership of medical schools in Uganda so as to provide evidence-based data to inform the selection and development of competent leaders of medical schools in Uganda. The study also identified the roles of medical school deans and leadership training needs. In keeping with observations by Guest et al^34^, our study reached data saturation after the fifth interview. Three major categories of leadership roles for a medical school dean were identified: Academic roles, Administrative roles, and Professional roles; affirming Lee and Hoyle's^35^ observations that a dean is responsible for three core functions of “management, academic leadership and professional leadership”.

The key academic leadership roles identified for deans of medical schools in Uganda, such as delivering on the academic mission of the school with an accredited curriculum, research leadership, and advancement of the school have also been reported by Muriisa^6^ in assessing the role of leadership in university performance in Uganda. Similar findings have been reported by Hromas et al^36^ in a study with over 800 academic leaders from 41 medical schools in the US and by Bassaw^37^.

Administratively the dean was reported to be responsible for the strategic, financial and resources management of the school including human resources development as has also been reported by Bassaw^37^, Hromas et al 36 and Yukl^5^. Furthermore, and in agreement with Bassaw^37^, the study established that the dean was responsible for quality assurance and establishing collaborations and efficient communication within and with various external stakeholders.

From our study, a dean should be a medical or health expert, providing professional leadership in the community of practice and beyond. This is in agreement with findings by Black^7^, Hromas et al^36^ and Lee and Hoyle^35^. A dean is also expected to supervise practice and the training of other health professionals^38^.

Our study identified eleven (11) key leadership competencies grouped into three key categories as personality-related competencies to do with the individual's behaviour and attributes such as trustworthiness, honesty, humility and integrity; educational competencies acquired as a result of academic training and expertise such as research and IT skills, health professions education and medical expertise; and the broader organizational management competencies related to finance and resources management; strategic and change management and the work-related behaviours. These are similar to the components of Mumford et al's^39^ five component competency model, the leadership skills strataplex40 and findings by Palmer et al^41^. Furthermore, Gigliotti and Ruben^24^ observed that, “...a broad understanding of the higher education landscape, an array of organizational and leadership concepts and tools, and the professional and personal competencies necessary to translate these capabilities into practice on a routine basis” are vital for effective performance. The three key competency categories identified in this study also incorporate the elements of the Great Eight Competencies espoused by Bartram^42^,^43^.

Two of the three competency categories (personality-related competencies and organizational competencies) established in this study are also similar to those captured in the categorization by Lieff and Albert^44^ -intrapersonal (personality-related); interpersonal, organizational and systemic competencies (organizational competencies). The organizational management competencies such as strategic management and people management skills from our study are also part of Kouzes and Posner's^45^ “Five Practices of Exemplary Leadership.”

The findings from our study highlighting the importance of behaviours and attributes such as honesty, integrity, trustworthiness and humility as key competencies for effective performance of a dean and a leader in general have also been reported by other authors^9^,^12^,^20^,^22^. In a study involving 22 current and former medical school deans in the US, patience, flexibility; a systems approach and ability to handle complexity emerged as important competencies for success^9^.

From the interviews it was found that a medical school dean should be a medical or health expert with skills in health professions education and research. This finding is in keeping with the theory of “expert leadership”^46^. The theory posits that leaders should poses the “technical expertise” and “industry experience” in the organization's core functions^46^. A similar view is expressed by other authors^8^,^20^,^24^ although Lobas^47^ challenges this view as a cottage industry mind-set.

Our study found that strategic management, communication and collaboration, and change management were major competencies required of a dean. Similarly, Kouzes and Posner^45^ and Senge et al^48^ have emphasized the importance of a systems approach to leadership which requires competencies in collaboration, advocacy and negotiation. Lobas^47^ further recommends that leaders in academic medicine should develop complex business leadership skills.

Competencies in communicating a compelling vision and implementing change within a dynamic environement have been cited by several authors^5^,^38^,^49^,^50^,^51^ as core competencies for effective organizational leadership. A dean deals with people from all walks of life including students, staff, faculty, senior university leadership and other stakeholders. Skills in people management a finding reported by Kouzes and Posner^45^ and Hayes et al^52^ are a requirement for effective deanship of a medical school.

In agreement with Muriisa^6^, Revere et al^53^ and Rich et al38, financial and resources management including resource mobilization featured as a key competency. Deans control resources and should therefore have entrepreneurial skills. Although some participants said that the dean should already possess the necessary competencies by the time they assume the position of dean, many authors^12^,^24^,^37^,^54^ support the need for training especially in leadership skills and financial and resources management.

## Limitations

There are only 12 medical schools, making a small study sample of 12 current deans interviewed as the primary respondents. However former deans that had been out of office for more than three years, or had served in higher positions such as college principal after deanship, were also enrolled to enrich the data and to obtain a richer perspective. The criteria above helped mitigate the potential bias in the purposive sampling of former deans. In addition, the inclusion of former deans, provided an enriched insight into the roles and key leadership competencies for medical school deans. As argued by Hollenbeck et al^14^, leadership competencies are usually identified by the incumbents then cross-validated with other groups.

## Conclusions

We set out to investigate the leadership competencies for effective deanship of medical schools in Uganda as perceived by current and former deans of medical schools in Uganda; and to identify training needs. Deans are responsible for the academic, administrative and professional leadership of the medical school. The key competencies for successful deanship of a medical school in Uganda encompass personality, education/training and organizational management competencies. The identification, selection, development and assessment of medical school deans should take into account a broad range of leadership qualities and go beyond the academic training and qualifications to include personality, financial and resources management, and competencies in medical and health professions education.

The leadership of universities and medical schools should consider the training of current and prospective deans of medical schools especially in leadership and finance management. The key leadership competencies identified in our study can be developed through a deliberate and systematic effort to enhance the deans' performance and in turn the growth and competitive advantage of their medical schools.

Further research using other techniques and methods such as focused group interviews and mixed-methods; and exploring the perceptions of other university leaders including those under the dean level is needed to provide a more in-depth understanding of the key competencies for effective leadership of medical schools in Uganda.

## Previous Presentations

This work was part of the dissertation for the Master of Business Administration (Leading Innovations and Change) submitted to York St. John University in June, 2020
